# Addressing Psychosocial Disabilities Through Social Interventions for Individuals With Severe Mental Disorders

**DOI:** 10.7759/cureus.61762

**Published:** 2024-06-05

**Authors:** Shazia Tahira

**Affiliations:** 1 Psychology, Bahria University, Karachi, PAK

**Keywords:** psychosocial intervention, psychosocial support systems, psychiatric rehabilitation, social work, psychosocial functioning, bipolar disorder, depression, schizophrenia, mentally ill persons, mental disorders

## Abstract

Mental disorders are prevalent worldwide, often causing significant distress and impairment across various life domains. Furthermore, they may lead to psychosocial disabilities exacerbated by stigma, discrimination, and social exclusion that hinder full societal participation and frequently result in human rights violations denying access to education, work, high-quality health, and reproductive rights. Therefore, a comprehensive and coordinated response to mental health requires a biopsychosocial approach and the integration of holistic promotion, prevention, support, care, and rehabilitation. Effective interventions need to be recovery-focused and should include social interventions. This editorial discusses the social interventions that can be utilized to address psychosocial disabilities in individuals with severe mental disorders. There is a need for developing innovative strategies, tools, and digital solutions, the provision of psychoeducation and caregiver support, along with conducting recovery-oriented research and provider training. Furthermore, the focus should be more on strengths instead of pathology and on cultivating a mental health-promoting environment. This requires inclusive policies, increased advocacy to decrease stigma and promote human rights, redirecting funds to community-based services from long-stay mental hospitals, and a multisectoral collaboration between different sectors such as employment, education, health, housing, social, and judicial sectors to provide support across different life stages, facilitate access to human rights, and attain equal opportunities to help individuals with severe mental disorders reach their full potential and live a meaningful life.

## Editorial

Mental health is a state of well-being that helps individuals acknowledge their potential, manage life's stressors, learn well and perform effectively, and give back to their communities [1]. Mental disorders are clinically significant emotional, behavioral, or cognitive disturbances due to dysfunctional developmental, biological, or psychological processes that impact mental and behavioral functioning and may lead to distress and impairment in personal, educational, occupational, family, social, or other important areas of functioning. Furthermore, long-term mental disorders and mental impairments may lead to psychosocial disability when the affected individuals interact with several obstacles such as stigma, discrimination, marginalization, and exclusion that prevent them from fully and equally participating in society [1]. With a prevalence of approximately one in eight persons, mental disorders are highly frequent globally and are the major factor causing a number of years spent disabled worldwide, causing one in every six years lived with disability; in this regard, schizophrenia impacts around one in 200 adults, and acute schizophrenia is the most impairing and debilitating health condition, as a person with acute schizophrenia is predicted to have only 20% of the health and functionality of a healthy person, whereas severe depressive episodes are ranked fifth in causing impaired health state and residual schizophrenia is ranked tenth [1]. Furthermore, individuals with severe mental disorders such as schizophrenia and bipolar disorder mostly have earlier deaths, 10 to 20 years before the general population, often due to physical illnesses that can be prevented [1].

Psychosocial disability is a disability involving activity limitations in individuals with mental disorders due to external social barriers [1]. Factors that impact psychosocial disability and provide protection from it may include individual factors, family and community factors, and structural factors. Individual factors include physical health, innate and acquired abilities, skills, and habits for managing emotions, activities, obligations, and social relationships, and a sense of capability, mastery, and self-worth. Family and community factors are related to immediate surroundings, such as the individual’s physical safety and security, their social and economic considerations, their interaction with parents, family, friends, partners, and coworkers, and their possibilities to participate in meaningful activities, earn a living, and get social support. Structural factors are related to an individual’s broader environmental, geopolitical, and sociocultural surroundings and include policies and systems related to social justice and equality, economic security, social protection, equal access to resources and services, access to necessities, infrastructure, environmental quality, and social stability [1].

The World Health Organization Comprehensive Mental Health Action Plan (2013-2030) highlights the prevalence of inappropriate confinement and homelessness among individuals with mental disorders, leading to their vulnerability and marginalization [2]. According to this plan, discrimination and stigmatization often result in human rights violations, denying affected individuals social, economic, and cultural rights, including education, work, high-quality health, and reproductive rights. These individuals may face unhygienic living conditions, neglect, abuse, and degrading treatment. Civil and political rights are frequently denied to them, including the right to personal liberty, the ability to vote, to engage fully and effectively in public life, to marry and start a family, and to use their legal capacity on matters that affect them, such as their care and treatment. The WHO Comprehensive Mental Health Action Plan (2013-2030) identifies a severe shortage of all mental health workers, especially those trained in the use of psychosocial interventions, in low- and middle-income countries; furthermore, limited policies and legislation on mental health are observed in low-income countries compared to high-income countries. When the plan was presented, annual per-person expenditure on mental health worldwide was less than US$2, and in low-income countries, it was less than US$ 0.25; furthermore, despite their link to human rights violations and suboptimal health outcomes, 67% of the world's annual spending on mental health was received by stand-alone mental hospitals [2]. According to the WHO Comprehensive Mental Health Action Plan (2013-2030), a comprehensive and coordinated response to mental health requires a biopsychosocial approach and integration of holistic promotion, prevention, support, care, and rehabilitation, addressing the needs for both physical and mental health care and promoting the recovery of individuals with mental disorders of all ages in and between general health services and social services by means of care, treatment, and recovery plans that are guided by the needs of the individual service user and, when appropriate, with the involvement of caregivers and families. The plan advocates redirecting funds to community-based services from long-stay mental hospitals. According to this plan, community-based mental health services should adopt a recovery-based approach, prioritizing individuals' goals and aspirations. Key elements include listening to individuals and treating them as equal partners. A multisectoral approach involving partnerships between different public and private sectors such as employment, education, health, housing, social, judicial, and other sectors is recommended to support individuals across different life stages and facilitate access to human rights like education, employment, health, and housing, as well as engagement in meaningful pursuits and community activities, through the provision of educational opportunities, return-to-work programs, primary and emergency health care, home care and support, supported housing, outreach services, community programs, and community-based rehabilitation [2].

The WHO-AIMS (Assessment Instrument for Mental Health Systems) cross-national analysis of 42 countries, including low-income countries, lower-middle-income countries, and upper-middle-income countries, analyzed collaborative links between mental health and other health and non-health sectors that can play an important role in the prevention and treatment of mental disorders [3]. According to that analysis, the links for collaboration between other departments and the government's Department of Mental Health were found to be weaker in low-income countries compared to lower- and upper-middle-income countries. The Department of Mental Health's collaborative links with the Department of Education were found in 60% of upper-middle-income countries, 74% of lower-middle-income countries, and only 54% of low-income countries. The proportion of schools with mental health professionals was 0% in low-income countries, 8% in lower-middle-income countries, and 16% in upper-middle-income countries. Links between mental health departments and employment departments were found in 42% of low-income countries, 36% of lower-middle-income countries, and 20% of upper-middle-income countries. In 10% of the countries, legislation protecting patients from discrimination at work, such as lower wages or dismissal only due to a mental disorder, was present and enforced, whereas 33% of countries had such provisions without enforcement and 57% of countries did not have any legislative provision; furthermore, 80% of upper-middle-income countries, 42% of lower-middle-income countries, and 30% of low-income countries were found to have such provisions. Legislative employment provisions such as employers' legal obligation to recruit a specific percentage of individuals with disabilities existed with enforcement in only 5% of countries; in 40% of countries, such provisions existed without enforcement; and in 55% of countries, no such provisions existed. Also, 60% of upper-middle-income countries, 46% of lower-middle-income countries, and 38% of low-income countries had such provisions. Housing sector collaborative links were found to be weak in all 42 participating countries. Financial or legislative provisions for housing were found in one-third of the participating countries but enforced only in 50% of those countries. As much as 40% of upper-middle-income countries, 38% of low-income countries, and 13% of lower-middle-income countries were found to have such provisions. Financial or legislative provisions to protect against housing discrimination were found in only 14% of the reporting countries, including 23% of low-income countries, 20% of upper-middle-income countries, and 8% of lower-middle-income countries [3].

A systematic review by Killaspy et al. (2022), which identified studies mostly conducted in high-income countries such as the United States, Australia, Canada, or European countries, discovered that despite many efforts over the past few decades to develop services that can enable people with severe mental health problems to integrate into their local communities and consideration of housing, employment services, and integrative community services, these individuals continue to be among the most marginalized in society [4]. This review found that, in Australia, barely one-third of those suffering from psychotic disorders had a job, and those who did were over twice as likely to report feeling lonely as the general population. Individuals with severe mental disorders are more likely to experience poverty, substandard housing, and unemployment, and these factors have a detrimental effect on their ability to integrate into society and worsen their mental health issues. Addressing the social impact of severe mental disorders is essential to breaking the cycle of social exclusion, but improving social outcomes for individuals with severe mental disorders is a complex challenge involving stigma, deficiencies in mental health systems, and the symptoms of the illness itself. While positive symptoms like delusions and hallucinations contribute to the diagnosis, negative symptoms and cognitive deficits hinder social inclusion by impacting social skills and motivation. Individual recovery, focusing on meaningful relationships and social roles, is crucial. Despite their importance and greater potential for effectiveness, social interventions have been overshadowed by pharmaceutical and psychological therapies in research and practice. Implementing social interventions is challenging compared to pharmacological therapies and psychological therapies due to their complexity and the need for support from various stakeholders. This study found strong evidence in favor of family psychoeducation, the Individual Placement and Support model of supported employment, and the Housing First model of supported housing; growing evidence supporting recovery colleges, peer-led and supported programs, and other community participation-promoting initiatives; and the significance of contextual elements and the necessity of local modifications when importing interventions from outside. In addition, this study found that supported employment and housing, such as Individual Placement and Support in the United Kingdom, United States, and Australia, and Housing First in the United States and Canada due to government support and policies, have widespread adoption, while family interventions have had difficulty being put into practice [4].

In 2013, globally, 45% of countries reported the presence of plans and policies for mental health that complied with human rights standards; therefore, the WHO Comprehensive Mental Health Action Plan's initial goal was to raise that percentage to 80% by 2020, and in 2019, there was an extension of the deadline to 2030, but until 2020, only 51% of countries had mental health plans and strategies in alignment with human rights standards, and only 21% of countries had such policies actually being implemented, including only 3% of low-income countries [1]. Overall, while progress has been made, the WHO Comprehensive Mental Health Action Plan 2013-2030 targets are still far from being met by the global community, highlighting the need for continued efforts and innovative solutions.

According to the World Mental Health Report 2022, mental health care is gradually shifting from institutionalization and hospital-based care to community-based approaches, but worldwide the progress is very slow, the multisectoral approach in its truest sense is utilized very rarely, and significant challenges remain in achieving comprehensive care [1]. In most countries, there are still insufficient financial and human resources dedicated to mental health, and mental health accounts for a very small portion of health expenditures worldwide. Globally, only 2% of the health budget is allocated to mental health, and for more than half of the world population, there is only one psychiatrist to treat 200,000 individuals, whereas mental health practitioners trained in psychosocial interventions are even fewer [1]. Furthermore, these inadequate resources mostly end up in psychiatric hospitals. In this regard, psychiatric hospitals continue to receive 66% of the expenditure spent on mental health globally and more than 70% in middle-income countries, and even now, community mental health services are rarely present in low-income countries [1]. The range of available interventions is mostly limited and mostly includes only biomedical-based care. The hospitals are frequently located far from the majority of people's places of residence and seldom offer the care that patients require; therefore, the treatment gap in the case of severe mental disorders is an astounding 90% in some countries [1]. Regarding education, employment, housing, and legal support, globally, less than 45% of countries provide any of these supports, and only 24% provide all these types of support; for example, housing support is provided by 36% of countries globally and only 4% of low-income countries [1]. Additionally, less than 5% of research funding is directed toward low- and middle-income countries, with the majority of mental health research being conducted in high-income countries [1]. But even in high-income countries, pharmacological interventions are much more commonly available compared to psychosocial interventions, with the provision of pharmacological interventions at the primary care level in 71% of high-income countries compared to psychosocial interventions in 34% of high-income countries [1]. These findings show that there is still much need for improvement, especially in countries with limited financial and human resources, which may include lower- and middle-income countries. In this regard, the following social intervention approaches may be useful to manage psychosocial disabilities in individuals with severe mental disorders.

Psychosocial rehabilitation

Psychosocial rehabilitation is a series of activities aimed at enhancing the functioning and minimizing the disability of individuals with mental disorders [1]. Community-based psychosocial rehabilitation helps individuals reach their full potential and integrate into society. In order to enable people with mental disorders to lead fulfilling lives in the community, psychosocial rehabilitation entails both changing the environment and enhancing people's competencies. Support that is recovery-focused, person-centered, and grounded in human rights is crucial. Furthermore, meeting the needs of people for social inclusion and independent living in accordance with their wishes and preferences, as well as successful deinstitutionalization, depends on the community's ability to provide psychosocial rehabilitation activities. Community mental health teams and centers play a significant role in providing psychosocial rehabilitation by combining outreach, livelihood, and routine activities. Peer group discussions, housing support, training in independent living and social skills, education and career support, social support network-building activities, and leisure activities are some examples of these activities. Another aspect of psychological rehabilitation is helping people access social and health services, such as welfare benefits or housing [1].

Strengths-based approach

A strengths-based approach shifts the emphasis from the shortcomings of individuals with mental illnesses to their strengths, qualities, and assets [5]. Rather than utilizing the conventional medical model, which places emphasis on pathology and failures in individuals with mental illnesses, the strength-based approach enables practitioners to recognize that each person possesses a distinct set of strengths and abilities that they can depend on to overcome challenges. Despite having mental diseases, mental health recovery is a personal journey towards a more meaningful life. By emphasizing an individual's abilities, giving them the confidence to start their recovery path, and assisting them in moving closer to mental health recovery, the strengths-based approach is consistent with the idea of mental health recovery. People's skills are highlighted instead of their flaws, ailments, or challenges. Mental health problems are accepted as a typical aspect of life. Positive aspects of individuals, such as their assets, dreams, aspirations, and hobbies, are stimulated and developed. Understanding one's strengths can help prevent or decrease the effects of stress, disease, and disorders [5].

Fostering empowerment and supporting multisectoral recovery plans

Healthcare professionals can foster empowerment by including, involving, encouraging, and empowering people with severe mental disorders and psychosocial disabilities to take an active role in their mental health treatment and care [2]. Furthermore, healthcare professionals can support multisectoral recovery plans by advocating for inclusion, providing support, and connecting individuals with severe mental disorders with resources and services that meet their needs and preferences, such as education, vocational training, work and employment, livelihood opportunities and support, social welfare, health care, housing, and legal support [2].

Developing innovative strategies, tools, and digital solutions

Innovative strategies or tools for the care and self-help of individuals with mental disorders may be created and implemented, such as the enhanced utilization and application of mobile and electronic technologies, possibly as a component of a stepped-care approach, with the least intensive treatment as the initial step and stepping up to more intensive services only when required, as well as the establishment of operational procedures, policies, and capacity for the remote provision of services, such as telehealth, and, when possible, the utilization of digital health solutions to assist practitioners in the provision of treatment and care [2]. 

Psychoeducation and caregiver support

To promote health and well-being, individuals with mental disorders and their caregivers and families may be educated about the causes, effects, and options for treatment and recovery of mental disorders and psychosocial disabilities, along with behaviors that may lead to a healthy lifestyle [2]. Furthermore, there may be a provision of caregiver skills training for caregivers and access to multidisciplinary community and in-home support services such as occupational therapy, physical therapy, early childhood development support, nutritional support, education support, and housing support [2].

Recovery-focused research and provider training

The scope of research may be enhanced to identify needs and assess the efficiency, viability, implementation, and expansion of services, programs, and initiatives, especially those that are recovery-focused and support human rights; additionally, research may be conducted to determine local perceptions and manifestations of mental distress in diverse cultural contexts; detrimental practices such as discrimination and violations of human rights; protective practices such as traditional rituals and customs and social supports; and help-seeking practices such as traditional healers; and the viability, acceptability, and effectiveness of interventions for promotion, prevention, treatment, care, and recovery [2]. Furthermore, training and opportunities for awareness-building for health professionals and social workers may be fostered to promote recovery-focused, culturally competent care, treatment, and support [2].

Inclusive policies and reshaping the environment

In order to better safeguard mental health and prevent mental disorders, we must alter and reshape the physical, social, and economic attributes of environments in homes, workplaces, schools, and the broader community [1]. Everybody needs to be given an equal chance to flourish in these settings and achieve the best possible degree of mental health and well-being. Understanding the structural and social determinants of mental health and taking action to lower risks, foster resilience, and remove obstacles preventing people with mental health conditions from fully participating in society are the fundamental components of reshaping environments for improved mental health. This involves incorporating mental health prevention and promotion with social and health services. Furthermore, it involves strategic initiatives and macro-level policies such as enforcing laws and regulations or putting in place suitable support systems to address disadvantages, defend human rights, and make certain that everyone has fair, unbiased, and equal access to opportunities, infrastructure, and services. The majority of the necessary strategies and interventions cannot be provided only by the health sector. However, by advocating, promoting, initiating, and, when necessary, facilitating multisectoral coordination and collaboration for mental health, the health sector plays a significant role in allowing and enabling action. In order to guarantee that people with mental disorders have access to high-quality treatments and are empowered, governments must give mental health a high priority in their agendas and policies. In addition to promoting integrated treatment and care approaches, caregivers and care providers, including mental health professionals, general health workers, community workers, and family members, must provide dignified, respectful, and courteous care that promotes and facilitates independence. Furthermore, non-governmental organizations, employers, academia, and stakeholders from civil society can supplement the government and care providers by campaigning for inclusive policies and promoting awareness. By working together and prioritizing mental health, we can create a future where everyone's well-being is enhanced, lives are improved, and suffering is decreased [1].

Although 11 years have passed since the WHO Comprehensive Mental Health Action Plan and only six years remain to achieve the targets before the extended deadline of 2030, the current situation is still far from perfect, and there is still much need for improvement in the management of patients with severe mental disorders, especially in countries where insufficient financial and human resources are dedicated to mental health, such as lower and middle-income countries. To reach these goals, Table 1 highlights the major factors that impact psychosocial disability, along with the existing gaps, suggested social interventions for improvement, and the challenges in implementation.

**Table 1:** Individual, family and community, and structural factors impacting psychosocial disability, current gaps, suggested social interventions, and implementation challenges

Individual factors	Family and community factors	Structural factors	Current gaps	Suggested social interventions	Implementation challenges
Physical health	Physical safety and security	Social justice and equality	Inadequate policies and laws	Inclusive policies and laws	Discrimination
Emotional and social skills	Parents and family	Equal access to resources	Insufficient research and lack of providers	Recovery-focused research and provider training	Lack of budget
Sense of capability and self-worth	Social networks and social supports	Economic security and social protection	Limited services and lack of multisectoral social support	Independence-facilitating care, multisectoral recovery plans, and family psychoeducation	Overshadowed by pharmacotherapy

Severe mental disorders impact not just the body or mind but also other areas of life and may lead to psychosocial disabilities; therefore, effective interventions that can have a greater impact need to be recovery-focused and should include social interventions and social supports, depending on the needs of the patients. There is a need for developing innovative strategies, tools, and digital solutions, the provision of psychoeducation and caregiver support, along with conducting recovery-oriented research and provider training. Psychiatry and mental health services need to be reoriented toward the community, and in addition, there should be a provision of social interventions at the primary health level. Furthermore, whether in the community or healthcare facilities, the provision of interventions must be in accordance with human rights. Additionally, the focus should be more on strengths instead of pathology and on cultivating a mental health-promoting environment, which requires inclusive policies, increased advocacy to decrease stigma and promote human rights, and multisectoral collaboration to attain equal opportunities to help individuals with severe mental disorders live up to their role as a social being and an active entity in society and reach their full potential to live a meaningful life.
